# Health Self-Management Behaviors as a Bridge Between Electronic Health Literacy and Health-Related Quality of Life: Cross-Sectional Study From China

**DOI:** 10.2196/74056

**Published:** 2025-08-13

**Authors:** Meihui Zhang, Shunyu Tao, Xin Ge, Hongying Zhang, Zhongqing Xu, Ruijie Gong, Yujie Liu, Chen Xu, Suping Wang, Yong Cai

**Affiliations:** 1 School of Public Health, Shanghai Jiao Tong University School of Medicine, Shanghai, China; 2 Public Health Research Center, Tongren Hospital, Shanghai Jiao Tong University, Xianxia Road, No.1111, Shanghai, 200336, China, 86 13611677244; 3 Nursing Department, Renji Hospital Affiliated to Shanghai Jiao Tong University School of Medicine, Shanghai, China; 4 Department of General Practice, Tongren Hospital, Shanghai Jiao Tong University School of Medicine, Shanghai, China; 5 Institute of Community Medicine, China Academy of Hospital Development, Shanghai Jiao Tong University, Shanghai, China; 6 Shanghai Xuhui Center for Disease Prevention and Control, Shanghai, China; 7 Shanghai Jiao Tong University School of Medicine Shanghai, Shanghai, China; 8 Institute of Medical Education, China Academy of Hospital Development, Shanghai Jiao Tong University, Shanghai, China

**Keywords:** electronic health literacy, health-related quality of life, health self-management behaviors, mediating effect, general population

## Abstract

**Background:**

Electronic health literacy (eHL) has been increasingly associated with health-related quality of life (HRQoL). However, the underlying mechanisms, especially in the general population, remain insufficiently explored.

**Objective:**

This study aimed to investigate the mediating role of health self-management behaviors (HSMB) in the relationship between eHL and HRQoL.

**Methods:**

A cross-sectional study was conducted in Shanghai, China, from October to December 2022. Participants were recruited via convenience sampling from 7 community health service centers. Data were collected through an online survey platform Wenjuanxing. Validated scales, including the eHL Scale, the adults’ health self-management skill rating scale, and the 12-item short form health survey were used to measure eHL, HSMB, and HRQoL, respectively. The HRQoL was summarized into the physical component summary (PCS) and the mental component summary (MCS). Correlation analysis, multivariate linear regression with stepwise backward selection, and mediation analysis were performed to explore the relationships among eHL, HSMB, PCS, and MCS, with adjustments for sociodemographic and health-related covariates.

**Results:**

Among the 2364 participants recruited from urban, periurban, and rural areas, eHL scores varied significantly by demographic characteristics. Positive correlations among eHL, HSMB, PCS, and MCS were observed, with Spearman correlation coefficients ranging from 0.24 to 0.46 (*P*<.001). Multivariate analysis showed that eHL was significantly positively associated with PCS (*R*^2^=0.14, 95% CI 0.09‐0.18, *P*<.001) and MCS (*R*^2^=0.23, 95% CI 0.17‐0.28, *P*<.001). Mediation analysis indicated that eHL had a significant direct (PCS: β_c_=.18, 95% CI 0.13‐0.23, *P*<.001; MCS: β_c_=.32, 95% CI 0.25‐0.38, *P*<.001) and an indirect effect on HRQoL through HSMB (PCS: β_c’_=.11, 95% CI 0.09‐0.14, *P*<.001; MCS: β_c’_=.14, 95% CI 0.10‐0.17, *P*<.001).

**Conclusions:**

This study demonstrated a positive association between eHL and HRQoL, with HSMB acting as a partial mediator among the general population in Shanghai. Targeted interventions should be implemented to improve eHL and HSMB.

## Introduction

Health-related quality of life (HRQoL), a crucial indicator in the comprehensive assessment of health outcomes, refers to individuals’ self-perceived functioning and well-being in physical, mental, and social domains of life [1]. Evaluating HRQoL helps identify individuals with illnesses or impaired physical and mental function [2], making it a vital concept in health management [3]. Improving HRQoL is essential for advancing health promotion and preventive medicine, aligning with the goals of the Healthy China 2030 Initiative, which seeks to enhance population health and build a sustainable, equitable health system [4].

With the advancement of internet technology, an increasing number of individuals seek health information through digital platforms such as websites, mobile apps, and social media, making the internet a significant medium for spreading health information [5], encouraging informed decision-making [6], and providing support to improve people’s HRQoL [7]. In this context, the concept of electronic health literacy (eHL) has emerged, which was defined as the ability of individuals to seek, find, understand, and appraise health information from electronic resources and apply the knowledge to address or solve health problems [8]. The World Health Organization (WHO) has also recognized the importance of eHL in achieving the sustainable development goals, particularly in strengthening health promotion and disease prevention worldwide through improvements in the quality, accessibility, and affordability of health services [9].

Previous studies have shown positive associations between eHL and greater accessibility to digital or virtual health care services [10,11], better behavioral (eg, health-promoting behaviors, medication adherence, and self-care) and cognitive (eg, health knowledge and health decision-making) outcomes [12], as well as self-rated health status and psychological well-being [13]. However, only a few studies have focused on the association between eHL and HRQoL. Although these studies have found an association between eHL and HRQoL, these findings are predominantly derived from specific populations such as older adults aged 60 years and older [14], young adults with type 2 diabetes [15], and college students [16]. Notably, there is a paucity of research examining this association within the general population. Given the escalating prevalence of subhealth conditions within the general population in China [17], the enhancement of eHL and HRQoL at the population level has become a critical public health priority, which has significant implications for reducing both health care system burdens and associated economic costs.

Health self-management behaviors (HSMB), a fundamental component of health self-management (HSM) ability, refer to the systematic actions individuals take to manage their health, including dieting, exercising, and dealing with diseases [18]. Distinct from general health-promoting behaviors, which encompass broader implications like health-related attitudes or knowledge change, HSMB emphasizes structured and sustained practices aimed at managing health [18,19]. The concept of HSM was initially focused on individuals with chronic illnesses [20]; however, there is an increasing awareness of its importance for the healthy and young population, as it’s the most cost-effective way to prevent diseases and maintain health [21]. The positive correlation between eHL and HSM has been found among undergraduate nursing students [21], older adults [14], and patients with chronic diseases [22]. People with higher eHL demonstrated a better ability to make full use of e-health resources [23], thereby being more easily influenced by health-promoting information and tending to adopt healthy behaviors. Furthermore, the association between HSM and HRQoL has also been reported in previous studies. A study conducted among patients with coronary heart disease found that better HSMB was significantly associated with better HRQoL [24]. Another study showed that self-management strategies can improve the HRQoL in patients with chronic disease [25].

Consequently, HSMB may mediate the relationship between eHL and HRQoL. However, only a limited number of studies have explored the potential mechanism for the relationship between eHL and HRQoL. A cross-sectional study conducted among Chinese older adults found that health-promoting behaviors mediated the relationship between eHL and HRQoL [14]. Another study also found a mediating role of self-management in the correlation between health literacy and HRQoL in patients with diabetic peripheral neuropathy [26]. Nonetheless, these 2 studies focused on specific populations and had certain differences in the measurement of the primary variables. In this study, we aimed to investigate the assumption that HSMB partially mediates the relationship between eHL and HRQoL, providing a theoretical basis for future projects or policies aiming to enhance national eHL, promote effective HSMB, and thereby improve the overall HRQoL of the population.

## Methods

### Study Design and Participants

From October to December 2022, a cross-sectional study using a multistage sampling method was conducted in Shanghai, China. Shanghai comprises 16 districts, which can be categorized into 7 central urban districts (eg, Xuhui, Huangpu), 3 periurban districts (eg, Minhang, Jiading), and 5 rural districts (eg, Jinshan, Chongming) based on urbanization level. At the time of the study, there were 247 community health centers distributed across these districts. To ensure representation, we first stratified the sampling framework by district type (urban, periurban, and rural). From each stratum, districts were randomly selected using a computer-generated random sequence, resulting in the inclusion of Xuhui (urban), Minhang (periurban), and Jinshan (rural). Within these 3 districts, 7 community health centers were selected via convenience sampling (2 in Xuhui, 3 in Minhang, and 2 in Jinshan), prioritizing feasibility and institutional collaboration. After identifying the community health service centers, we contacted the managers of each center to articulate the objective, content, and importance of this research. With their assistance, participants were recruited via convenience sampling during their visits to these centers throughout the study duration. The inclusion criteria included (1) being permanent residents of Shanghai aged 18 years or older and (2) being capable of providing informed consent. The exclusion criteria were (1) severe auditory or communicative impairments and (2) cognitive deficits impeding comprehension of the research content.

### Data Collection and Quality Control

Data were collected through an online survey platform Wenjuanxing (version 2024). Before the distribution of the questionnaires, a uniform training on the methodology, standardized operating procedures, ethical considerations, and electronic data collection protocols of this survey was provided to qualified researchers. Surveys were conducted in designated interview rooms at the community health centers, with trained researchers available on-site to assist participants in completing the questionnaire. Participants could complete the questionnaire either on their own mobile devices or on devices provided by the research team (iPad Pro, 2023 model). Furthermore, they could opt to fill out the questionnaire independently or with assistance from the researchers.

To ensure data quality, first of all, comprehensive quality control measures including real-time logical checks, automated response validation, and protected data encryption protocols were implemented through the platform’s integrated quality control system. Duplicate entries were avoided by WeChat account verification, allowing only one submission per account. Moreover, all eligible participants were informed of the objective, content, and estimated duration (approximately 20‐25 min) of this research before the survey to guarantee their full comprehension and voluntary participation. In addition, they were also informed that the survey was anonymous, and all collected data would be kept confidential and used solely for research purposes, ensuring the reliability of data collected. Ultimately, a total of 2372 participants completed the questionnaires, of which 2364 passed the quality assessment, resulting in an overall validity rate of 99.7%. As all survey items were mandatory, no missing data were present in the collected responses.

### Measurements

#### eHL

The eHL levels were assessed using the Chinese version of eHealth Literacy Scale (eHEALS) [27], translated and adopted from the initial version developed by Norman and Skinner [28]. The Chinese version of eHEALS comprised three dimensions: the ability to use online health information and services, the ability to judge health information, and the ability to make decisions, totaling 8 items. These items rated on a 5-point Likert scale, with total scores ranging from 8 to 40. Higher scores indicated higher eHL levels. The eHEALS has been validated among patients with stroke [29], older adults above 55 years [30], and college students [31] in China, with Cronbach α coefficient ranging from 0.91 to 0.94. In this study, the Cronbach α coefficient was 0.98.

#### HRQoL

The HRQoL was measured using the 12-item Short Form Health Survey (SF-12) [32]. The scale comprised 8 quality of life domains, which were summarized into 2 indices: the physical component summary (PCS) describing the general physical health, physical functioning, role limitations due to physical health, body pain and vitality, and the mental component summary (MCS) describing the mental health, social functioning, and role limitations due to emotional problems. Both PCS and MCS were converted into standard scores ranging from 0 to 100, with higher scores indicating better HRQoL [33]. The SF-12 has been validated among the general Chinese population, with a Cronbach α coefficient of 0.98 [34]. In this study, the Cronbach α coefficient was 0.75.

#### HSMB

The HSMB was assessed using the Adults Health Self-Management Skill Rating Scale (AHSMSRS), designed by Chinese scholars in 2011 [35]. This scale consisted of 3 subscales: health self-management behaviors, health self-management environment, and health self-management perceptions. In this study, the health self-management behaviors subscale, which included 3D of diet self-management, exercise self-management, and disease coping, was used to measure the HSMB. The scores of the subscale were based on a 5-point Likert scale ranging from 14 to 70, with higher scores representing better HSMB. As a scale developed in the Chinese cultural context, the AHSMSRS demonstrated good reliability and validity among the Chinese population, with Cronbach α coefficient greater than 0.90 in many previous studies [21,35,36]. In this study, the Cronbach α coefficient of the subscale was 0.92.

#### Covariates

Information on covariates including sociodemographic characteristics, health status, health behaviors, psychological conditions, and quality of sleep was collected. Sociodemographic characteristics included gender, age, years of education, household income per month, job status, marital status, living situation, and regional location. Health status included BMI calculated as weight (kg) divided by height squared (m^2^) and number of self-reported chronic diseases diagnosed by doctors. Health behaviors included drinking and smoking status. Psychological conditions included depression assessed using the Patient Health Questionnaire-9 and anxiety assessed using the Generalized Anxiety Disorder 7-item Scale. Quality of sleep was measured using the Pittsburgh Sleep Quality Index, with a total score of 5 or lower indicating good sleep quality, and a score above 5 indicating poor sleep quality.

### Statistical Analysis

In the descriptive analysis, continuous variables were presented as means and SD, and categorical variables were presented as frequencies and percentages. The Kruskal-Wallis test was used to compare the 4 continuous variables including eHL score, HSMB score, PCS, and MCS across subgroups of baseline characteristics. In the correlation analysis, Spearman correlation coefficients between eHL, HSMB, PCS, and MCS were calculated and a heatmap was presented. Correlation coefficients of 0.1‐0.2 were regarded as poor, 0.3‐0.5 as fair, 0.6‐0.7 as moderate, and 0.8‐0.9 as very strong [37]. In the regression analysis, multivariate linear regression models for both PCS and MCS using stepwise backward selection approach were constructed to assess associations of eHL with HRQoL and HSMB with HRQoL. Regression coefficients with 95% CIs were calculated. Multicollinearity tests for covariates included in multivariate linear regression models for both PCS and MCS using stepwise backward selection approach were performed before mediation analysis. The mediation analysis was conducted under the assumption that HSMB partially mediates the association between eHL and HRQoL (both PCS and MCS). EHL was used as the independent variable, HSMB as the mediator variable, PCS, and MCS as the outcome variables. Point estimates were based on 5000 bootstrap samples, and 95% CIs were calculated. All statistical analyses were carried out using R (version 4.4.2, R Foundation for Statistical Computing). A 2-tailed *P* value of <.05 was considered statistically significant.

### Ethical Considerations

The research protocol received approval from the ethics committee of Xuhui District Center for Disease Control and Prevention (XHLL202205). All participants provided written informed consent before participation. Data were collected anonymously to ensure confidentiality. Participants who completed the questionnaire were provided with a standardized incentive package valued at 30 RMB (approximately US $4.2), comprising essential household items including a bath towel, a toothbrush, a toothpaste, and a bar soap.

## Results

### Baseline Characteristics

Among the 2364 participants enrolled in this study, 881 (37.3%) were male, 589 (24.9%) aged younger than 34 years, 1221 (51.7%) aged 35‐59 years, and 554 (23.4%) aged older than 60 years. The mean eHL score, HSMB score, PCS, and MCS of the whole participants were 31.59 (out of 40), 46.65 (out of 70), 44.73 (out of 100) and 50.46 (out of 100), respectively. Female had slightly higher eHL scores (*P*=.02) and MCS (*P*=.005), while male had higher PCS (*P*=.01). Participants aged over 60 years had lower eHL scores (*P*<.001) and PCS (*P*<.001), while they reported higher HSM scores (*P*<.001) and MCS (*P*<.001). In addition, the eHL scores showed significant differences across demographic characteristics including education levels, household income, job status, living situation, drinking and smoking status, number of diseases, depression and anxiety conditions, and sleep quality. The HSM scores were significantly different across education levels, job status, marital status, regional location, drinking and smoking status, depression and anxiety conditions, and sleep quality. The PCS showed significant differences across all demographic characteristics except living situation and drinking status. The MCS showed significant differences across all demographic characteristics except BMI, drinking status, and number of disease (Table 1).

**Table 1.** Baseline characteristics and comparisons by electronic health literacy (eHL), health self-management behaviors (HSMB), physical component summary (PCS), and mental component summary (MCS; n=2364).

Characteristic	Participants, n (%)	eH^a^ score		HSMB^b^ score		PCS^c^		MCS^d^	
		Mean (SD)	*P* value	Mean (SD)	*P* value	Mean (SD)	*P* value	Mean (SD)	*P* value
All participants		31.59 (6.64)		46.65 (10.25)		44.73 (7.44)		50.46 (9.40)	
Gender			.02		.68		.01		.005
Male	881 (37.3)	31.11 (6.99)		46.49 (11.11)		45.31 (7.14)		49.69 (9.84)	
Female	1483 (62.7)	31.87 (6.41)		46.74 (9.71)		44.38 (7.59)		50.92 (9.09)	
Age			<.001		<.001		<.001		<.001
≤34 years	589 (24.9)	31.58 (7.60)		43.71 (11.65)		46.59 (6.76)		47.98 (9.31)	
35‐59 years	1221 (51.7)	31.98 (6.30)		46.78 (9.94)		45.32 (7.06)		51.05 (9.03)	
≥60 years	554 (23.4%)	30.73 (6.21)		49.48 (8.33)		41.46 (7.94)		51.81 (9.80)	
Years of education			<.001		.01		<.001		.04
≤9 years	322 (13.6)	30.14 (7.43)		47.57 (10.81)		43.23 (7.99)		49.79 (10.18)	
10‐12 years	493 (20.9)	32.17 (6.55)		46.28 (10.27)		45.53 (7.16)		50.32 (9.17)	
≥13 years	1549 (65.5)	30.70 (6.13)		47.19 (9.76)		43.20 (7.57)		51.36 (9.52)	
Household income per month (US $)			<.001		.16		<.001		<.001
≤696.7	472 (20.0)	29.95 (7.51)		46.31 (10.08)		43.87 (7.50)		49.81 (9.56)	
696.7‐1393.0	772 (32.7)	31.07 (6.48)		45.98 (11.75)		44.34 (7.45)		49.04 (10.09)	
1393.1‐2786.1	704 (29.8)	32.70 (5.88)		47.41 (9.57)		44.99 (7.59)		51.69 (8.68)	
≥2786.2	416 (17.6)	32.51 (6.65)		46.73 (9.79)		46.32 (6.78)		51.21 (9.14)	
Employment status			<.001		<.001		<.001		<.001
Employed	1553 (65.7)	32.05 (6.82)		45.63 (10.77)		46.05 (6.79)		49.70 (9.27)	
Unemployed	811 (34.3)	30.71 (6.21)		48.58 (8.85)		42.19 (7.96)		51.91 (9.47)	
Marriage			.24		.002		<.001		<.001
Never married	312 (13.2)	31.71 (7.41)		44.70 (11.77)		46.77 (6.78)		47.49 (9.32)	
Married	1931 (81.7)	30.60 (7.28)		46.21 (10.83)		43.54 (8.30)		50.56 (9.80)	
Divorced or widowed	121 (5.1)	31.63 (6.47)		46.99 (9.91)		44.47 (7.44)		50.94 (9.30)	
Living situation			.02		.43		.86		<.001
Living alone	211 (8.9)	30.37 (7.6)		45.69 (12.44)		44.71 (7.85)		48.27 (9.58)	
Not living alone	2153 (91.1)	31.7 (6.53)		46.74 (10.01)		44.73 (7.40)		50.68 (9.35)	
Regional location			.78		.005		<.001		.004
Outer suburbs	949 (40.1)	31.47 (7.19)		45.70 (11.31)		45.96 (6.97)		49.79 (9.21)	
Inner suburbs	787 (33.30)	31.69 (6.40)		47.10 (9.66)		44.24 (7.45)		50.72 (9.67)	
Central urban	628 (26.6)	31.63 (6.06)		47.50 (9.13)		43.48 (7.84)		51.17 (9.27)	
BMI			.34		.25		.007		.15
Underweight	103 (4.4)	31.31 (6.55)		46.67 (9.83)		44.09 (7.67)		50.70 (9.39)	
Normal	1376 (58.2)	31.62 (6.74)		46.78 (10.51)		45.14 (7.34)		50.53 (9.33)	
Overweight	715 (30.3)	31.96 (6.60)		45.2 (10.29)		44.70 (7.70)		48.20 (10.16)	
Obesity	170 (7.2)	32.20 (6.24)		46.34 (9.83)		44.09 (6.98)		50.29 (9.36)	
Drinking status			<.001		.03		.13		.26
Never	1916 (81.1)	29.15 (7.55)		44.32 (10.24)		43.64 (5.37)		49.98 (9.81)	
≤2 times / week	355 (15.0)	31.81 (6.55)		46.86 (10.18)		44.69 (7.68)		50.59 (9.42)	
≥3 times / week	93 (3.9)	31.01 (6.76)		46.11 (10.54)		45.24 (6.50)		49.88 (9.14)	
Smoking status			<.001		.002		.004		<.001
Never	2045 (86.5)	29.14 (7.37)		43.89 (11.34)		42.76 (5.93)		44.90 (8.46)	
≤2 times/week	218 (9.2)	31.80 (6.56)		46.87 (10.22)		44.82 (7.54)		50.74 (9.36)	
≥3 times/week	101 (4.3)	30.72 (6.76)		45.81 (9.78)		44.79 (7.07)		50.44 (9.34)	
Number of diseases			<.001		.06		<.001		.67
0	1547 (65.4)	32.06 (6.74)		46.30 (10.75)		46.40 (6.76)		50.37 (9.29)	
1	555 (23.5)	29.63 (6.86)		47.79 (10.40)		36.38 (8.53)		49.85 (9.45)	
2	178 (7.5)	30.97 (6.18)		47.39 (9.34)		42.92 (7.11)		50.72 (9.60)	
3	84 (3.6)	30.33 (6.67)		46.78 (8.19)		39.76 (7.42)		50.74 (9.68)	
Depression			<.001		<.001		<.001		<.001
No	1076 (45.5)	33.04 (6.89)		49.81 (10.30)		46.86 (7.30)		55.11 (8.19)	
Yes	1288 (54.5)	30.37 (6.18)		44.01 (9.44)		42.95 (7.08)		46.58 (8.54)	
Anxiety			<.001		<.001		<.001		<.001
No	1347 (57.0)	32.85 (6.63)		48.98 (9.86)		46.12 (7.61)		54.59 (8.06)	
Yes	1017 (43.0)	29.90 (6.28)		43.55 (9.93)		42.89 (6.79)		45.00 (8.18)	
Sleep Quality			<.001		.01		<.001		<.001
Poor	205 (8.7)	29.15 (7.02)		45.10 (9.62)		38.34 (7.29)		45.23 (9.74)	
Good	2159 (91.3)	31.82 (6.56)		46.79 (10.30)		45.34 (7.17)		50.96 (9.21)	

a eHL: Electronic health literacy score; range 8-40, with higher scores indicating better electronic health literacy levels.

b HSMB: health self-management behaviors score; range 14-70, with higher scores indicating better health self-management behaviors.

c PCS: Physical Component Summary; range 0-100, with higher scores indicating better health-related quality of life.

d MCS: Mental Component Summary; range 0-100, with higher scores indicating better health-related quality of life.

### Correlation Between EHL, HSMB, PCS, and MCS

**Figure 1.**
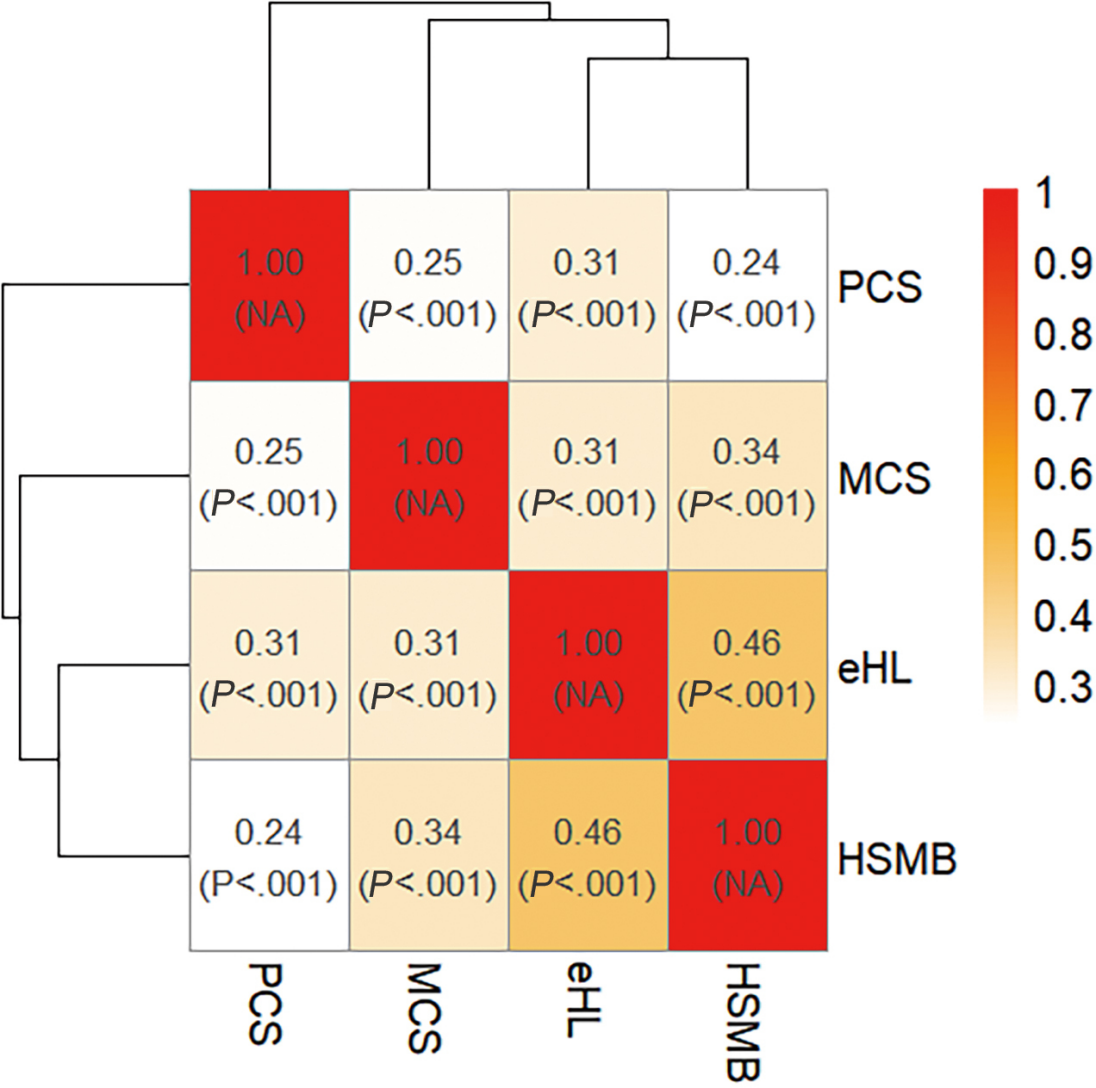
Spearman correlation coefficients heatmap between eHL, HSMB, PCS, and MCS (n=2364). eHL: electronic health literacy; HSMB: health self-management behaviors; MCS: mental component summary; PCS: physical component summary.

In the correlation analysis conducted among eHL, HSMB, PCS, and MCS, significant positive correlations were observed between each pair of variables, with Spearman correlation coefficients ranging from marginally poor-to-fair to fair (all *P*<.001). The strongest correlation was observed between eHL and HSMB (*r*=0.46, fair). EHL showed fair correlations with both PCS (*r*=0.31) and MCS (*r*=0.31). HSMB exhibited a fair correlation with MCS (*r*=0.34) and a marginally poor-to-fair correlation with PCS (*r*=0.24). The interrelationship between PCS and MCS was also poor-to-fair (*r*=0.25). The observed correlations, while modest in magnitude, demonstrated significant associations across all hypothesized pathways, thereby satisfying the necessary assumptions for subsequent mediation analysis. Figure 1 presented a heatmap illustrating the correlations among the four variables, with deeper colors indicating stronger correlations.

### Associations of EHL With HRQoL and HSMB With HRQoL

In the multivariate linear regression analysis for PCS, 12 variables were identified as significantly associated with PCS using stepwise backward selection approach and were included in the final model, including eHL, HSMB, gender, age, job status, marital status, regional location, BMI, number of diseases, depression, and anxiety conditions, and sleep quality. Both eHL and HSMB (both as continuous variables) showed significantly positive associations with PCS after adjusting for other selected variables, with regression coefficients of 0.14 (95% CI 0.09‐0.18, *P*<.001), 0.10 (95% CI 0.07‐0.13, *P*<.001), respectively. In the multivariate linear regression analysis for MCS, eleven variables were identified and included in the final model, including eHL, HSMB, gender, age, household income, job status, drinking and smoking status, depression and anxiety conditions, and sleep quality. Similar to PCS, the significantly positive associations of eHL with MCS and HSMB with MCS were also observed after adjusting for other selected variables, with regression coefficients of 0.23 (95% CI 0.17‐0.28, *P*<.001), 0.09 (95% CI 0.05‐0.12, *P*<.001), respectively (Tables2  3). The results of multicollinearity tests indicated no significant correlation among covariates included in the final multivariate linear regression models for both PCS and MCS (the variance inflation factor for all the factors was <5) (Table s1-s2 in Multimedia Appendix 1). The full multivariate linear regression models for both PCS and MCS before stepwise backward selection were presented in Tables S3-S4 in Multimedia Appendix 1.

**Table 2.** Multivariate linear regression models for physical component summary (PCS) using stepwise backward selection approach (n=2364).

Selected variables	*R*^2^ (95% CI)	*P* value
eHL^a^ (as continuous variable)	0.14 (0.09 to 0.18)	<.001
HSMB^b^ (as continuous variable)	0.10 (0.07 to 0.13)	<.001
Gender (compared with male)		
Female	−1.13 (−1.68 to −0.57)	<.001
Age (compared with ≤34 years)		
35‐59 years	−0.82 (−1.56 to −0.08)	.03
≥60 years	−1.86 (–3.04 to −0.69)	.002
Job (compared with employed)		
Unemployed	−0.92 (−1.74 to −0.11)	.03
Marriage (compared with never married)		
Married	−0.90 (−1.80 to -0.01)	.047
Divorced or widowed	−0.41 (−1.85 to 1.03)	.58
Regional location (compared with outer suburbs)		
Inner suburbs	0.04 (–0.63 to 0.71)	.90
Central urban	0.84 (0.16 to 1.52)	.02
BMI (compared with underweight)		
Normal	0.28 (–0.99 to 1.56)	.67
Overweight	−0.18 (−1.51 to 1.16)	.80
Obesity	−0.88 (–2.45 to 0.70)	.28
Number of diseases (compared with 0)		
1	−2.14 (–2.83 to –1.45)	<.001
2	−4.07 (–5.17 to –2.97)	<.001
3	−7.22 (–8.71 to –5.72)	<.001
Depression (compared with no)		
Yes	−2.38 (–3.11 to –1.65)	<.001
Anxiety (compared with no)		
Yes	−0.82 (–1.56 to –0.08)	.03
Sleep Quality (compared with poor)		
Good	3.57 (2.62 to 4.52)	<.001

a eHL: electronic health literacy.

b HSMB: health self-management behaviors.

**Table 3.** Multivariate linear regression models for mental component summary (MCS) using stepwise backward selection approach (n=2364).

Selected variables	*R*^2^ (95% CI)	*P* value
eHL^a^ (as continuous variable)	0.23 (0.17 to 0.28)	<.001
HSMB^b^ (as continuous variable)	0.09 (0.05 to 0.12)	<.001
Gender (compared with male)		
Female	1.08 (0.36 to 1.81)	.004
Age (compared with ≤34 years)		
35‐59 years	1.51 (0.74 to 2.27)	<.001
≥60 years	0.42 (–0.81 to –1.66)	.50
Household income per month (compared with US $696.7‐1393.0)		
≤US $696.7	0.70 (−0.18 to –1.59)	.12
US $1393.1‐2786.1	1.44 (0.54 to –2.35)	.002
≥US $2786.2	1.14 (0.11 to –2.18)	.03
Job (compared with employed)		
Unemployed	1.95 (0.98 to –2.91)	<.001
Drinking status (compared with never)		
≤2 times/week	1.14 (0.17 to –2.10)	.02
≥3 times/week	1.28 (−0.42 to –2.99)	.14
Smoking status (compared with never)		
≤2 times/week	−2.80 (−4.41 to −1.19)	.001
≥3 times/week	0.78 (−0.42 to –1.97)	.20
Depression (compared with no)		
Yes	−2.71 (–3.60 to −1.83)	<.001
Anxiety (compared with no)		
Yes	−5.82 (−6.71 to −4.93)	<.001
Sleep Quality (compared with poor)		
Good	2.77 (1.63 to –3.91)	<.001

a eHL: electronic health literacy.

b HSMB: health self-management behaviors.

### Mediating Role of HSMB in the Relationship Between EHL and HRQoL

**Figure 2.**
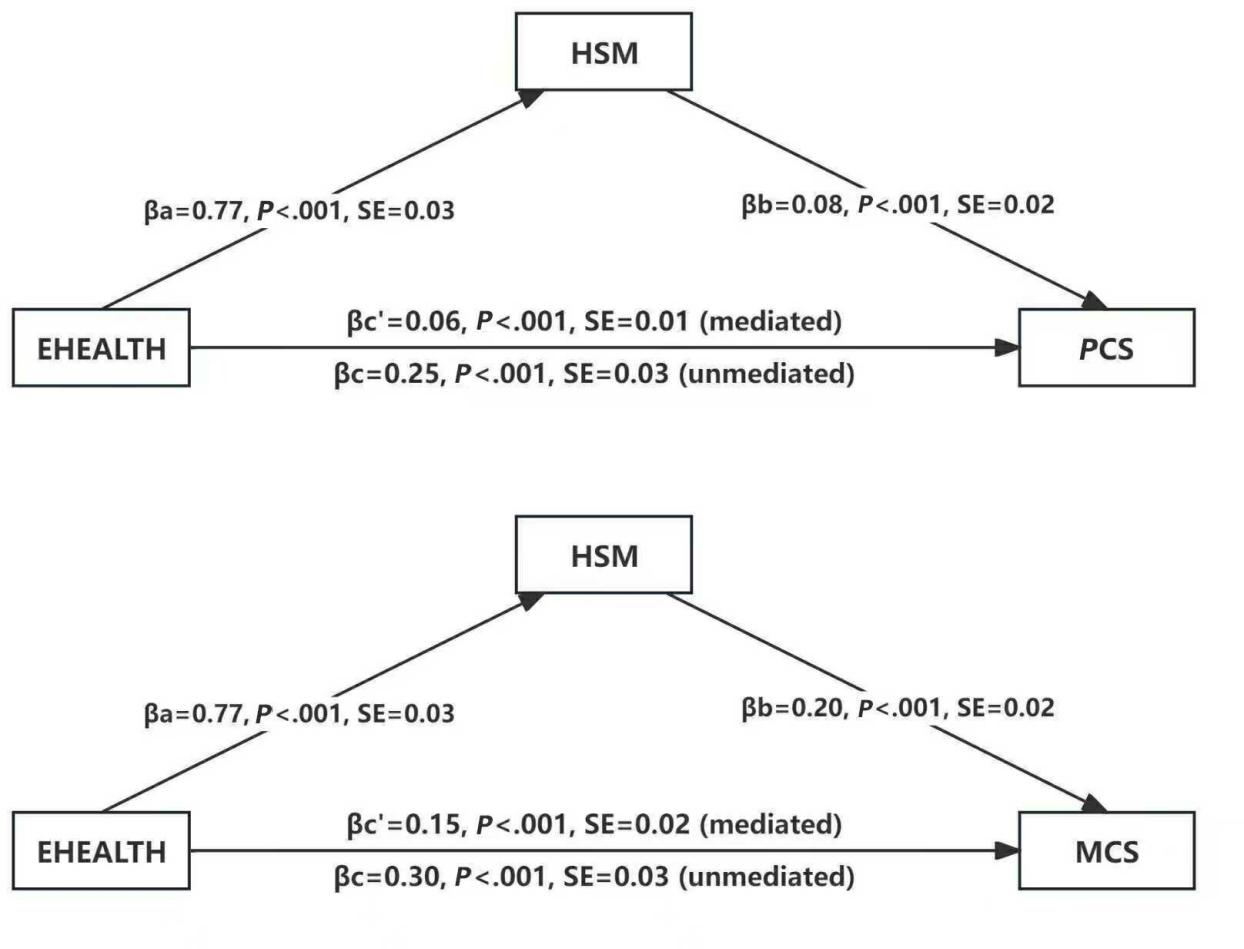
Bootstrap-mediating effect of HSMB on the associations of eHL with PCS and MCS (n=2364). eHL: electronic health literacy; HSMB: health self-management behaviors; MCS: mental component summary; PCS: physical component summary.

Figure 2 displayed the results of mediation analysis examining the role of HSMB in the relationship between eHL and HRQoL. After adjusting for regular sociodemographic covariates including gender, age, education levels, household income, job status, marital status, living situation, and regional location. The influence of eHL (independent variable) on HSMB (mediator variable) was statistically significant (β_a_=.80, 95% CI 0.74‐0.87, *P*<.001). In addition, HSMB demonstrated significant effects on both PCS (β_b_=0.14, 95% CI 0.11‐0.17, *P*<.001) and MCS (β_b_=.17, 95% CI 0.12‐0.21, *P*<.001; outcome variables). The direct effects of eHL on PCS (β_c_=0.18, 95% CI 0.13‐0.23, *P*<.001) and MCS (β_c_=.32, 95% CI 0.25‐0.38, *P*<.001) were both statistically significant. The indirect effects of eHL on PCS (β_c’_=.11, 95% CI 0.09‐0.14, *P*<.001) and MCS (β_c’_=.14, 95% CI 0.10‐0.17, *P*<.001) were also both statistically significant. The mediating effect accounted for 38.14% and 29.87% of the total effect for PCS and MCS, respectively. These findings indicated that HSMB partially mediated the relationship between eHL and HRQoL. The full results of mediation analysis were presented in Table S5-S6 Multimedia Appendix 1. In sensitivity analysis adjusting for covariates selected by the backward selection method, the total, direct, and indirect effects remained significant, and the results were presented in Table S7-S8 Multimedia Appendix 1.

## Discussion

### Principal Findings

In the current cross-sectional study conducted in Shanghai, China, we investigated the relationship between eHL and HRQoL and explored the mediating role of HSMB. The findings demonstrated a significant positive association between eHL and HRQoL, with HSMB acting as a partial mediator in this relationship within the Chinese population. These results underscored the potential mechanism by which eHL may enhance HRQoL through the adoption of HSMB. The insights gained from this research may offer valuable evidence for developing targeted interventions to improve HRQoL among Chinese adults, particularly in light of the rapid expansion of internet-based health information platforms and the growing emphasis on eHL within the Healthy China 2030 initiative.

### Comparison to Previous Work

The mean eHL score of participants was 31.59, which was consistent with findings from a previous study conducted in Poland (mean score was 30.69) [38], while significantly higher than that reported for the general population in Hubei Province, China (mean score was 24.7) [39]. This indicated that in Shanghai, one of the most developed cities in China, the eHL level of residents was on par with that of populations in developed nations and ranked highly nationwide. The results also revealed that advanced age, lower income levels, and unemployment status were significantly associated with low eHL levels, aligning with findings from prior studies conducted in mainland China [14], Hong Kong [40], and the United States [41]. These disparities highlighted the challenges faced by vulnerable populations, including older adults who struggle to adapt to rapidly evolving digital technologies, and low-income or unemployed individuals who have limited access to digital devices and educational resources. To address these inequities, interventions such as community-based eHL training programs, aligning with the Healthy China 2030 goal of improving eHL and reducing disparities [4], could be implemented. These programs, inspired by WHO’s global strategy on eHL [9], would focus on improving digital skills and access to reliable health information, particularly for those vulnerable populations. Integrating eHL education into primary health care services could empower individuals to better manage their health through digital tools, thereby reducing the digital divide and promoting health equity.

The study demonstrated a significant positive association between eHL and HRQoL. This suggested that individuals with lower eHL levels were more likely to experience impaired physical health and poorer mental well-being. This finding aligned with previous research that identified similar positive associations between eHL and HRQoL in older adults aged 60 and older [14], young adults with type 2 diabetes [15], and college students [16]. However, while existing evidence has largely been derived from studies focusing on vulnerable or clinical populations, the present investigation has extended this understanding to the general population. Further supporting evidence comes from 2 studies conducted in distinct cultural contexts. A cross-sectional study of Korean older adults revealed that higher eHL was significantly associated with better self-perceived health status among community-dwelling individuals aged 65 years and older [42]. Another study conducted in a United States cohort of chronic obstructive pulmonary disease patients demonstrated that while eHL was positively correlated with lung-specific HRQoL [43]. However, although the outcomes in these 2 studies were highly related to HRQoL, they still differed in scope and conceptualization. To the best of our knowledge, this was the first study conducted among the general population in China to examine the association between eHL and HRQoL, offering a more inclusive perspective on the role of eHL in promoting population health. In light of the growing burden of subhealth conditions in China [17], these findings highlighted the potential value of population-level strategies to improve eHL and HRQoL. In line with the National Health Literacy Promotion Three-Year Action Plan (2024‐2027) launched by the National Health Commission of China [44], targeted interventions such as releasing high-quality health science works to address key health topics and populations, and organizing health science competitions to encourage innovation in health education could be implemented to effectively enhance eHL.

This study also found that HSMB partially mediated the relationship between eHL and HRQoL, revealing the underlying mechanisms between eHL and HRQoL. This finding supported earlier evidence which indicated that health-promoting behaviors served as a mediator in the relationship between eHL and HRQoL among Chinese older adults [14], and self-management served as a mediator in the relationship between HL and HRQoL among patients with diabetic peripheral neuropathy [26]. However, this study extended the existing evidence by exploring the mediating role of HSMB, distinct from previously measured constructs in the association between eHL and HRQoL within a broader, more generalizable population. Moreover, the eHL, HSMB, and HRQoL were measured with high reliability, as evidenced by their high Cronbach α coefficients, underscoring the robustness and internal consistency of the findings. However, it is noteworthy that the HSMB measurement scale was developed within the Chinese cultural context. Cautions are needed when applying it to other cultural contexts. For example, the scale’s emphasis on “scientifically allocating breakfast or lunch or dinner proportions (good breakfast, full lunch, and light dinner)” reflects traditional Chinese dietary principles, which may not align with meal patterns in Western cultures. Similarly, in low-resource health care settings, items like “using professional guidance for exercise selection” could face implementation challenges due to limited access to medical resources. Such cultural differences highlight the need for cross-cultural validation of HSMB construct to ensure the generalizability of mediation mechanisms across diverse populations.

The mediation effect of HSMB can be explained by the Information-Motivation-Behavioral Skills model, which highlights the interplay of information, motivation, and behavioral skills in driving health behavior change [45]. In this study, high eHL empowered individuals to effectively acquire and use health-related information, equipping them with the knowledge necessary to manage their health. This enhanced informational capacity facilitated the development of HSMB. Through these mechanisms, HSMB mediated the relationship between eHL and HRQoL, transforming eHL into actionable behaviors that ultimately improved health outcomes. These findings underscored the need for systematic integration of eHL training and HSMB promotion. Community health service center is an ideal platform to effectively translate these insights into practice. First, standardized eHL and HSMB education modules with content adapted for different population groups should be developed. For example, design simplified digital interfaces for older adults and more advanced information evaluation techniques for younger patients. These modules could be delivered by trained community health workers during routine health consultations and chronic disease management visits. Second, mobile health applications and various media platforms could support these programs by enabling real-time self-monitoring and facilitating communication between patients and providers. Third, trained community health workers could provide competency-based instruction in facilitating interactive small-group sessions and individualized counseling during clinical encounters, with particular emphasis on adapting communication for varying health literacy levels. To ensure sustainability, these programs should be incorporated into existing primary care performance evaluation systems and supported through dedicated funding mechanisms within national health initiatives.

Furthermore, the coefficients in the mediation analysis hold significant implications, thus warranting further interpretation. The results indicated that each 1-point increase in eHL score produced a 0.29-point and 0.11-point enhancement in PCS in total and through the HSMB-mediated pathway alone, respectively. For MCS, each 1-point increase in eHL score produced a 0.46-point and 0.14-point enhancement in total and through the mediation effect, respectively. A study conducted on patients with left-main coronary artery disease or 3-vessel disease across 18 countries in North America and Europe established clinically significant cutoff values of 45.5 for PCS and 52.3 for MCS, which were associated with improved 10-year survival outcomes following coronary revascularization [46]. In comparison, the mean PCS and MCS of the whole participants in the present study were 44.73 and 50.46, respectively, indicating modest but meaningful gaps of 0.77 and 1.84 points, respectively, to reach these prognostic thresholds. A 4-point enhancement in eHL scores, which is achievable through targeted digital literacy programs, would fill the gap in both PCS and MCS.

The findings of this study also hold significant implications for global health policy, particularly in the context of the rapid digital transformation of health care systems [47]. By embedding eHL as a core component of national and international health strategies, equitable distribution of digital health benefits can be reached worldwide. This is particularly vital in low- and middle-income countries, where the development of supportive infrastructure remains insufficient to the transformation of digital health information [48]. Simultaneously, promoting HSMB is equally critical as demonstrated by its mediating role in this study. Enhancing HSMB globally requires a multifaceted approach that integrates digital tools, education, and policy support. For instance, mobile health applications can be leveraged to deliver personalized self-management strategies, particularly for chronic disease prevention and management. International collaboration supported by organizations such as the WHO is crucial to advancing eHL and HSMB as key drivers of improved HRQoL globally. By sharing best practices and resources, such efforts can bridge infrastructure gaps and ensure equitable access to health resources and promote the adoption of HSMB.

### Strengths and Limitations

This study has several notable strengths. First, we investigated the mediating role of HSMB in the relationship between eHL and HRQoL within a general population. By extending the existing evidence beyond vulnerable or clinical populations, this study provided a more comprehensive understanding of the mechanism through which eHL influenced HRQoL. Second, the use of validated and reliable measurement tools including the eHEALS, the SF-12, and the AHSMSRS, ensured the robustness and internal consistency of the findings. Third, this study rigorously controls for a wide range of sociodemographic covariates in the mediation analysis, minimizing the confounding factors and strengthening the validity of the observed associations between eHL, HSMB, and HRQoL.

However, several limitations should be acknowledged. First, the cross-sectional design of the study precluded the establishment of causal relationships between eHL, HSMB, and HRQoL. While we have used rigorous statistical methods including adjustments for key covariates and sensitivity analyses, longitudinal studies are needed to confirm the temporal sequence and causality of these associations. Second, although the study sample was from various areas of Shanghai, one of China’s most developed metropolises, which could not fully represent the broader Chinese population, particularly those in less developed regions. As in some less developed provinces, the eHL level of population is much lower, which could alter the observed relationships. Though stratified sampling across urban, periurban, and rural districts mitigated the influence of socioeconomy, future research should include participants from a wider range of geographic and socioeconomic backgrounds and explore the moderation effect of socioeconomic factors on the relationships among eHL, HSMB, and HRQoL in these populations to enhance external validity. Third, while the study identified HSMB as a mediator, other potential mediators, such as social support or health service usage, were not explored. We have collected extensive covariates covering demographics, lifestyle factors, physical health, and mental health to minimize omitted variable bias, but future research still should consider a broader range of mediating factors to provide a more comprehensive understanding of the pathways through which eHL influences HRQoL. Finally, the reliance on self-reported data might introduce information bias. However, we have implemented multiple safeguards to enhance reliability, including the use of validated instruments, strict quality control methods, and researcher-supervised data collection procedures.

### Conclusion

This study demonstrated a positive association between eHL and HRQoL, with HSMB acting as a partial mediator in this relationship within the general population in Shanghai. Enhancing eHL and promoting HSMB are crucial for improving HRQoL, particularly in the context of growing burden of subhealth conditions and the rapid expansion of internet-based health information platforms. Targeted interventions should be implemented to improve eHL and HSMB among the general population, especially for vulnerable populations, such as older adults and individuals with lower socioeconomic status.

## Supplementary material

10.2196/74056 Multimedia Appendix 1 Additional tables.

## Abbreviations

AHSMSRS: Adults Health Self-Management Skill Rating Scale
eHEALS: eHealth Literacy Scale
eHL: electronic health literacy
HRQoL: health-related quality of life
HSMB: health self-management behaviors
HSM: health self-management
MCS: Mental Component Summary
PCS: Physical Component Summary
SF-12: 12-item Short Form Health Survey
WHO: World Health Organization

## References

[1] Kaplan RM, Hays RD (2022). Health-Related Quality of Life Measurement in Public Health. Annu Rev Public Health.

[2] Xie L, Mo PKH (2024). A 3-Wave Longitudinal Study of eHealth Literacy and Older People’s Health-Related Quality of Life in China: The Mediating Role of General Self-Efficacy. J Am Med Dir Assoc.

[3] van Leeuwen KM, van Loon MS, van Nes FA (2019). What does quality of life mean to older adults? A thematic synthesis. PLoS ONE.

[4] China TSCotPsRo (2019). Action of Healthy China (2017–2030).

[5] Wang C, Wu X, Qi H (2021). A Comprehensive Analysis of E-Health Literacy Research Focuses and Trends. Healthcare (Basel).

[6] Chen YY, Li CM, Liang JC, Tsai CC (2018). Health Information Obtained From the Internet and Changes in Medical Decision Making: Questionnaire Development and Cross-Sectional Survey. J Med Internet Res.

[7] Liu L, Wu F, Tong H, Hao C, Xie T (2021). The Digital Divide and Active Aging in China. Int J Environ Res Public Health.

[8] Norman CD, Skinner HA (2006). eHealth Literacy: Essential Skills for Consumer Health in a Networked World. J Med Internet Res.

[9] (2019). Regional Action Agenda on Harnessing E-Health for Improved Health Service Delivery in the Western Pacific.

[10] Yao R, Zhang W, Evans R, Cao G, Rui T, Shen L (2022). Inequities in Health Care Services Caused by the Adoption of Digital Health Technologies: Scoping Review. J Med Internet Res.

[11] Budhwani S, Fujioka J, Thomas-Jacques T (2022). Challenges and strategies for promoting health equity in virtual care: findings and policy directions from a scoping review of reviews. J Am Med Inform Assoc.

[12] Xie L, Zhang S, Xin M, Zhu M, Lu W, Mo PKH (2022). Electronic health literacy and health-related outcomes among older adults: A systematic review. Prev Med.

[13] Milanti A, Chan DNS, Parut AA, So WKW (2023). Determinants and outcomes of eHealth literacy in healthy adults: A systematic review. PLoS ONE.

[14] Li S, Cui G, Yin Y, Wang S, Liu X, Chen L (2021). Health-promoting behaviors mediate the relationship between eHealth literacy and health-related quality of life among Chinese older adults: a cross-sectional study. Qual Life Res.

[15] Jang Y, Yang Y (2025). Effects of e-health literacy on health-related quality of life in young adults with type 2 diabetes: Parallel mediation of diabetes self-efficacy and self-care behaviors. Appl Nurs Res.

[16] Li S, Cui G, Zhou F (2022). The Longitudinal Relationship Between eHealth Literacy, Health-Promoting Lifestyles, and Health-Related Quality of Life Among College Students: A Cross-Lagged Analysis. Front Public Health.

[17] Xue Y, Huang Z, Liu G (2020). Association analysis of Suboptimal health Status: a cross-sectional study in China. PeerJ.

[18] Zhao Q, Huang F (2011). Developm entand the reliability and validity test of the rating scale of health self-management for adults. Chinese Journal of Modern Nursing.

[19] Yu H (2024). Understanding Health-Promoting Behaviors and Influential Factors in Schizophrenia Patients. Nutrients.

[20] Allegrante JP, Wells MT, Peterson JC (2019). Interventions to Support Behavioral Self-Management of Chronic Diseases. Annu Rev Public Health.

[21] Zhang S, Wang W, Wu S (2024). Analysis of the mediating effect between ehealth literacy and health self-management of undergraduate nursing students’ mental health literacy. BMC Nurs.

[22] Shiu LS, Liu CY, Lin CJ, Chen YC (2023). What are the roles of eHealth literacy and empowerment in self-management in an eHealth care context? A cross-sectional study. J Clin Nurs.

[23] Rangraz Jeddi F, Nabovati E, Mobayen M (2023). Health care needs, eHealth literacy, use of mobile phone functionalities, and intention to use it for self-management purposes by informal caregivers of children with burns: a survey study. BMC Med Inform Decis Mak.

[24] Zhang Y, Yan F, Jiang W (2019). Relationship between self-management behaviors and health-related quality of life among Chinese patients with coronary heart disease: A cross-sectional study. Contemp Nurse.

[25] Kang E, Kim S, Rhee YE, Lee J, Yun YH (2021). Self-management strategies and comorbidities in chronic disease patients: associations with quality of life and depression. Psychol Health Med.

[26] Gao Y, Yan K, Yan X, Xi N, Gao J, Ren H (2023). Correlation between health literacy and health-related quality of life in patients with diabetic peripheral neuropathy: The mediating role of self-management. Nurs Open.

[27] Shuai-jun G, Xiao-ming Y, Yu-ying S, Dan N, Xue-min L, Lu W (2013). Adaptation and evaluation of Chinese version of eHEALS and its usage among senior high school students. Chinese Journal of Health Education.

[28] Norman CD, Skinner HA (2006). eHEALS: The eHealth Literacy Scale. J Med Internet Res.

[29] He Y, Guo L, Zauszniewski JA (2023). A reliability and validity study of the electronic health literacy scale among stroke patients in China. Top Stroke Rehabil.

[30] Xie L, Mo PKH (2023). Comparison of eHealth Literacy Scale (eHEALS) and Digital Health Literacy Instrument (DHLI) in Assessing Electronic Health Literacy in Chinese Older Adults: A Mixed-Methods Approach. Int J Environ Res Public Health.

[31] Li S, Cui G, Kaminga AC, Cheng S, Xu H (2021). Associations Between Health Literacy, eHealth Literacy, and COVID-19-Related Health Behaviors Among Chinese College Students: Cross-sectional Online Study. J Med Internet Res.

[32] Ware JE, Kosinski M, Keller SD (1996). A 12-Item Short-Form Health Survey: construction of scales and preliminary tests of reliability and validity. Med Care.

[33] Ware J, Kosinski M, Keller S (2023). SF-12: how to score the SF-12 physical and mental health summary scales. 1995.

[34] Lam C, Wong C, Lam E, Lo Y, Huang W (2010). Population norm of Chinese (HK) SF-12 health survey-version 2 of Chinese adults in Hong Kong. Hong Kong Practitioner.

[35] Qiu-li Z, Fei-fei H (2011). Development and the reliability and validity test of the rating scale of health self-management for adults. Chinese Journal of Modern Nursing.

[36] Ren J, Zhang X, Zeng JH, Gao YX (2024). Influencing factors of self-management ability among dry eye patients in west China. Int J Ophthalmol.

[37] Fadahunsi KP, Wark PA, Mastellos N (2025). A Novel Framework to Assess Clinical Information in Digital Health Technologies: Cross-Sectional Survey Study. JMIR Med Inform.

[38] Burzyńska J, Rękas M, Januszewicz P (2022). Evaluating the Psychometric Properties of the eHealth Literacy Scale (eHEALS) among Polish Social Media Users. Int J Environ Res Public Health.

[39] Sun C, Meijer E, Chavannes NH (2025). eHealth literacy in the general population: a cross-sectional study in China. BMC Public Health.

[40] Guo Z, Zhao SZ, Guo N (2021). Socioeconomic Disparities in eHealth Literacy and Preventive Behaviors During the COVID-19 Pandemic in Hong Kong: Cross-sectional Study. J Med Internet Res.

[41] Nagori A, Keshvani N, Patel L, Dhruve R, Sumarsono A (2024). Electronic health Literacy gaps among adults with diabetes in the United States: Role of socioeconomic and demographic factors. Prev Med Rep.

[42] Kyaw MY, Aung MN, Koyanagi Y (2024). Sociodigital Determinants of eHealth Literacy and Related Impact on Health Outcomes and eHealth Use in Korean Older Adults: Community-Based Cross-Sectional Survey. JMIR Aging.

[43] Stellefson M, Paige SR, Alber JM (2019). Association Between Health Literacy, Electronic Health Literacy, Disease-Specific Knowledge, and Health-Related Quality of Life Among Adults With Chronic Obstructive Pulmonary Disease: Cross-Sectional Study. J Med Internet Res.

[44] (2024). Notice on the launch of the three-year action plan to improve national health literacy (2024-2027). China NHCo.

[45] Xu H, Wang J (2023). An information-motivation-behavioral skills model-based intervention for patients with epilepsy. Epilepsy Behav.

[46] Ono M, Serruys PW, Garg S (2022). Effect of Patient-Reported Preprocedural Physical and Mental Health on 10-Year Mortality After Percutaneous or Surgical Coronary Revascularization. Circulation.

[47] Stoumpos AI, Kitsios F, Talias MA (2023). Digital Transformation in Healthcare: Technology Acceptance and Its Applications. Int J Environ Res Public Health.

[48] Kaboré SS, Ngangue P, Soubeiga D (2022). Barriers and facilitators for the sustainability of digital health interventions in low and middle-income countries: A systematic review. Front Digit Health.

