# Supplementary material for: Health Self-Management Behaviors as a Bridge Between Electronic Health Literacy and Health-Related Quality of Life: Cross-Sectional Study From China
Source: J Med Internet Res. 2025 Aug 13;27:e74056. doi: 10.2196/74056 (PMC12349732; doi:10.2196/74056)
Supplement: Multimedia Appendix 1 [file jmir-v27-e74056-s001.docx]

Table s1. Multicollinearity test of covariates included in multivariate linear regression model for physical component summary (PCS) using stepwise backward selection approach

| **Variable** | **GVIF** | **Df** | **Adjusted_GVIF** |
| --- | --- | --- | --- |
| Gender | 1.118 | 1 | 1.058 |
| Age | 3.434 | 2 | 1.361 |
| Job | 2.302 | 1 | 1.517 |
| Marriage | 1.441 | 2 | 1.096 |
| Location | 1.183 | 2 | 1.043 |
| BMI | 1.144 | 3 | 1.023 |
| Ndisease | 1.583 | 3 | 1.080 |
| Depression | 2.072 | 1 | 1.439 |
| Anxiety | 2.073 | 1 | 1.440 |
| Sleep | 1.107 | 1 | 1.052 |
| eHL | 1.436 | 1 | 1.198 |
| HSMB | 1.495 | 1 | 1.223 |

Abbreviations: Ndisease, number of diseases; eHL, electronic health literacy; HSMB, health self-management behaviors

Table s2. Multicollinearity test of covariates included in multivariate linear regression model for mental component summary (MCS) using stepwise backward selection approach

| **Variable** | **GVIF** | **Df** | **Adjusted_GVIF** |
| --- | --- | --- | --- |
| Gender | 1.317 | 1 | 1.148 |
| Age | 2.288 | 2 | 1.230 |
| Income | 1.117 | 3 | 1.019 |
| Job | 2.25 | 1 | 1.500 |
| Drinking | 1.402 | 2 | 1.088 |
| Smoking | 1.388 | 2 | 1.085 |
| Depression | 2.073 | 1 | 1.440 |
| Anxiety | 2.067 | 1 | 1.438 |
| Sleep | 1.097 | 1 | 1.047 |
| eHL | 1.456 | 1 | 1.207 |
| HSMB | 1.492 | 1 | 1.221 |

Abbreviations: eHL, electronic health literacy; HSMB, health self-management behaviors

Table s3. Full multivariate linear regression model for physical component summary (PCS) (n= 2,364)

| **All Variables** | **RC [95%CI]** | ***P* value** |
| --- | --- | --- |
| **eHL (as continuous variable)** | 0.13[0.09 - 0.18] | <.001 |
| **HSMB (as continuous variable)** | 0.10[0.07 - 0.13] | <.001 |
| **Gender (compared with male)** |  |  |
| Female | -1.23[-1.85 - -0.60] | <.001 |
| **Age (compared with ≤ 34 years)** |  |  |
| 35-59 years | -0.84[-1.60 - -0.08] | .03 |
| ≥ 60 years | -1.73[-2.93 - -0.53] | .005 |
| **Years of education (compared with ≤ 9 years)** |  |  |
| 10-12 years | -0.28[-1.18 - 0.62] | .55 |
| ≥ 13 years | 0.00[-0.88 - 0.89] | .99 |
| **Household income per month (compared with ≤ 5000 yuan)** |  |  |
| 5000-9999 yuan | -0.65[-1.41 - 0.11] | .09 |
| 10000-19999 yuan | -0.45[-1.26 - 0.35] | .27 |
| ≥ 20000 yuan | 0.16[-0.77 - 1.09] | .74 |
| **Job status (compared with employed)** |  |  |
| Unemployed | -0.91[-1.74 - -0.07] | .03 |
| **Marriage (compared with never married)** |  |  |
| Married | -0.98[-1.93 - -0.03] | .04 |
| Divorced or widowed | -0.34[-1.79 - 1.11] | .65 |
| **Living situation (compared with living alone)** |  |  |
| Not living alone | -0.24[-1.27 - 0.79] | .65 |
| **Regional location (compared with outer suburbs)** |  |  |
| Inner suburbs | 0.10[-0.57 - 0.77] | .77 |
| Central urban | 0.92[0.21 - 1.64] | .01 |
| **BMI (compared with underweight)** |  |  |
| Normal | 0.35[-0.93 - 1.62] | .60 |
| Overweight | -0.12[-1.45 - 1.22] | .86 |
| Obesity | -0.80[-2.38 - 0.78] | .32 |
| **Drinking status (compared with never drinking)** |  |  |
| ≤ 2 times / week | 0.16[-0.64 - 0.96] | .70 |
| ≥ 3 times / week | 0.16[-1.26 - 1.58] | .83 |
| **Smoking status (compared with never smoking)** |  |  |
| ≤ 2 times / week | -1.13[-2.47 - 0.21] | .099 |
| ≥ 3 times / week | -0.26[-1.26 - 0.75] | .62 |
| **Number of diseases (compared with 0 disease)** |  |  |
| 1 | -2.09[-2.79 - -1.40] | <.001 |
| 2 | -3.99[-5.09 - -2.89] | <.001 |
| 3 | -7.10[-8.60 - -5.60] | <.001 |
| **Depression (compared with no)** |  |  |
| Yes | -2.33[-3.07 - -1.59] | <.001 |
| **Anxiety (compared with no)** |  |  |
| Yes | -0.81[-1.56 - -0.07] | .031 |
| **Sleep Quality (compared with poor)** |  |  |
| good | 3.55[2.59 - 4.50] | <.001 |

Table s4. Full multivariate linear regression model for mental component summary (MCS) (n= 2,364)

| **All Variables** | **RC [95%CI]** | ***P* value** |
| --- | --- | --- |
| **eHL (as continuous variable)** | 0.23[0.17 - 0.28] | <.001 |
| **HSMB (as continuous variable)** | 0.09[0.05 - 0.12] | <.001 |
| **Gender (compared with male)** |  |  |
| Female | 1.11[0.36 - 1.86] | .004 |
| **Age (compared with ≤ 34 years)** |  |  |
| 35-59 years | 1.10[0.18 - 2.01] | .02 |
| ≥ 60 years | 0.06[-1.38 - 1.51] | .93 |
| **Years of education (compared with ≤ 9 years)** |  |  |
| 10-12 years | 0.80[-0.28 - 1.89] | .15 |
| ≥ 13 years | 0.51[-0.56 - 1.57] | .35 |
| **Household income per month (compared with ≤ 5000 yuan)** |  |  |
| 5000-9999 yuan | 0.54[-0.38 - 1.46] | .25 |
| 10000-19999 yuan | 1.26[0.29 - 2.23] | .01 |
| ≥ 20000 yuan | 0.91[-0.21 - 2.03] | .11 |
| **Job status (compared with employed)** |  |  |
| Unemployed | 2.04[1.03 - 3.05] | <.001 |
| **Marriage (compared with never married)** |  |  |
| Married | 0.90[-0.25 - 2.05] | .12 |
| Divorced or widowed | 0.47[-1.28 - 2.23] | .60 |
| **Living situation (compared with living alone)** |  |  |
| Not living alone | -0.18[-1.43 - 1.06] | .77 |
| **Regional location (compared with outer suburbs)** |  |  |
| Inner suburbs | 0.08[-0.73 - 0.90] | .84 |
| Central urban | -0.23[-1.09 - 0.63] | .60 |
| **BMI (compared with underweight)** |  |  |
| Normal | 1.13[-0.41 - 2.67] | .15 |
| Overweight | 1.53[-0.08 - 3.14] | .06 |
| Obesity | 0.67[-1.23 - 2.58] | .49 |
| **Drinking status (compared with never drinking)** |  |  |
| ≤ 2 times / week | 1.17[0.21 - 2.13] | .02 |
| ≥ 3 times / week | 1.31[-0.40 - 3.02] | .13 |
| **Smoking status (compared with never smoking)** |  |  |
| ≤ 2 times / week | -2.73[-4.35 - -1.11] | .001 |
| ≥ 3 times / week | 0.84[-0.37 - 2.05] | .17 |
| **Number of diseases (compared with 0 disease)** |  |  |
| 1 | -0.47[-1.30 - 0.37] | .27 |
| 2 | -0.09[-1.42 - 1.23] | .89 |
| 3 | -0.95[-2.75 - 0.86] | .30 |
| **Depression (compared with no)** |  |  |
| Yes | -2.70[-3.59 - -1.81] | <.001 |
| **Anxiety (compared with no)** |  |  |
| Yes | -5.79[-6.68 - -4.90] | <.001 |
| **Sleep Quality (compared with poor)** |  |  |
| good | 2.70[1.55 - 3.86] | <.001 |

Table s5. Mediation analysis of health self-management behaviors (HSMB) on the associations between electronic health literacy (eHL) and physical component summary (PCS) adjusting for regular sociodemographic covariates (n= 2,364)

|  | **β** | **95%CI** | **SE** | ***P* value** |
| --- | --- | --- | --- | --- |
| **Total effect, c+c’** | 0.291 | (0.251, 0.332) | 0.021 | <.001 |
| **Direct effect, c** | 0.179 | (0.132, 0.230) | 0.025 | <.001 |
| **Indirect effect, c’** | 0.111 | (0.085, 0.140) | 0.014 | <.001 |
| **Mediated Proportion** | 38.14% | | | |

Table s6. Mediation analysis of health self-management behaviors (HSMB) on the associations between electronic health literacy (eHL) and mental component summary (MCS) adjusting for regular sociodemographic covariates (n= 2,364)

|  | **β** | **95%CI** | **SE** | **p-value** |
| --- | --- | --- | --- | --- |
| **Total effect, c+c’** | 0.452 | (0.398, 0.508) | 0.028 | <.001 |
| **Direct effect, c** | 0.317 | (0.253, 0.383) | 0.033 | <.001 |
| **Indirect effect, c’** | 0.135 | (0.100, 0.169) | 0.018 | <.001 |
| **Mediated Proportion** | 29.87% | | | |

Table s7. Mediation analysis of health self-management behaviors (HSMB) on the associations between electronic health literacy (eHL) and physical component summary (PCS) adjusting for covariates selected by the backward selection method (n= 2,364)

|  | **β** | **95%CI** | **SE** | ***P* value** |
| --- | --- | --- | --- | --- |
| **Total effect, c+c’** | 0.207 | (0.167, 0.246) | 0.020 | <.001 |
| **Direct effect, c** | 0.134 | (0.087, 0.181) | 0.024 | <.001 |
| **Indirect effect, c’** | 0.073 | (0.049, 0.097) | 0.012 | <.001 |
| **Mediated Proportion** | 35.27% | | | |

Table s8. Mediation analysis of health self-management behaviors (HSMB) on the associations between electronic health literacy (eHL) and mental component summary (MCS) adjusting for covariates selected by the backward selection method (n= 2,364)

|  | **β** | **95%CI** | **SE** | ***P* value** |
| --- | --- | --- | --- | --- |
| **Total effect, c+c’** | 0.302 | (0.244, 0.358) | 0.029 | <.001 |
| **Direct effect, c** | 0.235 | (0.176, 0.295) | 0.030 | <.001 |
| **Indirect effect, c’** | 0.067 | (0.036, 0.098) | 0.016 | <.001 |
| **Mediated Proportion** | 22.19% | | | |
